# *Lacticaseibacillus rhamnosus* OF44-15Ph10T Attenuates Bone Loss in Ovariectomized Rats

**DOI:** 10.3390/foods15213902

**Published:** 2026-10-30

**Authors:** Xiaoxia Sun, Jiajia Zhang, Yazhou Xu, Juntao Yu, Chenzhe Liu, Li Yang, Xiaobin Ruan, Shipei Chen, Qianqian Guan, Tao Huang, Tao Xiong, Mingyong Xie

**Affiliations:** 1 State Key Laboratory of Food Science & Resources, Nanchang University, No. 235 Nanjing East Road, Nanchang 330047, China; 2 School of Food Science and Technology, Nanchang University, No. 235 Nanjing East Road, Nanchang 330047, China; 3 International Institute of Food Innovation Co., Ltd., Nanchang University, Luozhu Road, Xiaolan Economic and Technological Development Zone, Nanchang 330200, China; 4 Jiangxi Institute for Drug Control, Jiangxi Province Engineering Research Center of Drug and Medical Device Quality, No. 1566 East Beijing Road, Qingshanhu District, Nanchang 330047, China

**Keywords:** osteoporosis, *Lacticaseibacillus rhamnosus*, gut microbiota, bone remodeling, Wnt/β-catenin signaling pathway

## Abstract

Estrogen deficiency compromises bone structure and mechanical competence, creating a need for interventions that preserve both. The bone-health effects of the probiotic candidate *Lacticaseibacillus rhamnosus* OF44-15Ph10T (OF44) have not been established. We evaluated OF44 in ovariectomized (OVX) rats, using micro-CT and biomechanical testing to assess skeletal outcomes and serum assays, histology, protein profiling, and 16S rRNA gene sequencing to characterize accompanying biological changes. Thirty female Sprague–Dawley rats were assigned to Sham, OVX, and OF44 groups (*n* = 10 per group; *n* = 6 per group for endpoint analyses). OF44 was administered by oral gavage at 5 × 10^8^ CFU/kg body weight/day for 90 days. Relative to OVX controls, OF44-treated rats had higher femoral bone mineral density (0.179 versus 0.132 g/cm^3^; +35.5%), trabecular bone volume fraction (10.10% versus 7.01%; +44.1%), and maximum load (+17.7%). These improvements were accompanied by a favorable serum bone turnover profile, fewer TRAP-positive osteoclasts, and increased serum IL-10. OF44 treatment also altered bone-remodeling-related protein expression and gut microbial composition, including enrichment of *Ruminococcaceae* and S24-7/*Muribaculaceae*. The convergent structural and mechanical outcomes provide strain-specific preclinical evidence for OF44 as a probiotic candidate for bone health under estrogen-deficient conditions.

## 1. Introduction

Bone is a dynamic tissue that undergoes continuous remodeling via the resorption of old bone and formation of new bone, processes mediated by osteoclasts and osteoblasts, respectively [1]. An imbalance in bone metabolism, characterized by excessive osteoclastic resorption over osteoblastic formation, can cause bone loss and promote the development of osteoporosis (OP) [2,3]. OP is the most common systemic skeletal disorder among elderly individuals and is characterized by reduced bone mass, deterioration of bone microarchitecture, and increased fracture risk [4]. The incidence of OP is influenced by multiple factors, including age, gender, endocrine status, and genetic predisposition. Among these, estrogen deficiency associated with menopause and aging plays a critical role. Studies indicate that more than 30% of postmenopausal women aged 50 years and older develop OP [5,6]. This disease severely impairs patients’ quality of life and places a substantial socioeconomic burden on society [7].

The gut microbiota, comprising microbial communities colonizing the host intestine, exerts a substantial influence on host health. It regulates bone metabolism through multiple mechanisms [8], including generating metabolic byproducts, interacting with host metabolic pathways, and modulating immune function. Accumulating evidence suggests that targeted regulation of the gut microbiota may serve as a safe and effective strategy for alleviating skeletal disorders, including OP and cartilage injury; among available approaches, probiotic intervention shows great potential [9,10,11]. The effects of probiotics on the gut microbiota have important therapeutic implications for the management of human and animal diseases. Previous studies have shown that estrogen deficiency-induced bone loss is often accompanied by gut microbial dysbiosis, including alterations in the Firmicutes/Bacteroidota ratio and changes in bacterial families involved in intestinal carbohydrate metabolism and host–microbiota interactions, such as Ruminococcaceae, Lachnospiraceae, and Muribaculaceae, as well as expansion of inflammation-associated taxa [12]. Probiotics are defined as live microorganisms that, when administered in adequate amounts, confer health benefits and favorably modulate bone metabolism [13]. Previous studies have demonstrated the potential of *Lacticaseibacillus rhamnosus* to alleviate estrogen deficiency-induced bone loss. Guo et al. reported that *L. rhamnosus* GG (LGG) improved bone microarchitecture and biomechanical properties in ovariectomized rats, accompanied by changes in gut microbiota composition, intestinal barrier function, and Th17/Treg balance [14]. Similarly, Sapra et al. demonstrated that *L. rhamnosus* administration attenuated bone loss in ovariectomized mice and was associated with immunomodulatory effects involving the Treg/Th17 balance [15]. The strain specificity of probiotic effects makes direct evaluation of OF44 essential for establishing its bone-health properties.

*Lacticaseibacillus rhamnosus* OF44-15Ph10T (OF44), isolated from a healthy female donor, exhibits tolerance to simulated gastrointestinal conditions and antimicrobial activity, supporting its evaluation as an orally administered probiotic candidate [16]. Its effects on estrogen deficiency-induced bone loss have not been established. Here, we tested whether OF44 preserves femoral trabecular structure and mechanical competence in ovariectomized rats. Serum bone turnover markers and histology linked the skeletal outcomes to remodeling-related changes, while protein profiling and 16S rRNA gene sequencing characterized accompanying bone-related protein expression and gut microbial composition. This design provides strain-specific evidence for the bone-health effects of OF44 and identifies biological associations for targeted investigation.

## 2. Materials and Methods

### 2.1. Preparation of Bacterial Suspensions

OF44 was obtained from BGI Precision Nutrition (Shenzhen, China) as a freeze-dried preparation and stored at −20 °C until use. Before administration, OF44 was activated by two consecutive 24-h subcultures at 37 °C in de Man, Rogosa, and Sharpe (MRS) medium (Hope Bio-Technology, Qingdao, China). The bacterial suspension used for oral administration contained 5 × 10^7^ CFU/mL, corresponding to a daily dose of 5 × 10^8^ CFU/kg body weight (BW) when administered at 10 mL/kg BW. The viable bacterial concentration was determined by plate counting before preparation of the gavage suspension.

### 2.2. Animals and Experimental Design

Three-month-old SPF female Sprague-Dawley rats were purchased from Zhuhai BesTest Biological Technology Co., Ltd. (Zhuhai, China). After quarantine and acclimatization, the 30 rats were assigned unique identification numbers and randomly allocated to a sham-operated (Sham) group (*n* = 10) and an ovariectomized (OVX) group (*n* = 20) using computer-generated random numbers. Rats in the sham-operated group underwent laparotomy without ovary removal, whereas rats in the OVX-operated group underwent bilateral ovariectomy. Both surgical procedures were performed under general anesthesia induced by intraperitoneal administration of a 1:1 (*v*/*v*) mixture of Zoletil 50 (50 mg/mL) and Sumianxin II (10 mg/mL). The mixture was administered at 0.14 mL/100 g body weight (BW), corresponding to doses of 35 mg/kg BW for Zoletil 50 and 7 mg/kg BW for Sumianxin II. Anesthetic depth was assessed using the pedal withdrawal reflex in response to toe pinching. Adequate anesthesia was generally maintained without additional administration, although supplemental anesthesia was required in a few animals. For intraoperative analgesia, lidocaine (2 mg/kg BW) was administered by local infiltration around the surgical incision. Postoperative analgesia was provided with carprofen (5 mg/kg BW), administered subcutaneously for two consecutive days.

Following surgery, rats were monitored twice daily (morning and evening) during the first three postoperative days and once daily thereafter. Clinical assessments included general behavior and activity, mobility and gait, posture, surgical incision condition (including signs of swelling, bleeding, infection, or wound dehiscence), coat condition and general appearance, food and water intake, and respiratory patterns. Body weight, food intake, and water intake were quantitatively recorded weekly. Predefined humane endpoints, as specified in the approved animal ethics protocol, included body weight loss exceeding 20% without recovery within 72 h, severe impairment of mobility with no response to gentle stimulation, persistent loss of appetite, inability to eat or drink due to weakness, moribund condition, and severe infection. No surgery-related adverse events, including deaths or wound infections, were observed, and all animals survived until the scheduled experimental endpoint without premature euthanasia. Five days after surgery, vaginal smears were performed daily for 3 consecutive days to confirm successful OVX modeling. After successful OVX modeling was confirmed, the OVX-operated rats were further randomly allocated to the model (OVX) group and the OF44-treatment (OF44) group (*n* = 10 per group) using the same computer-generated randomization procedure. The animal study was approved by the Institutional Animal Care and Use Committee (IACUC) of Zhuhai BesTest Biological Technology Co., Ltd. (approval no. IAC24W684; approval date: 30 October 2024). Rats were housed 5 per cage under controlled conditions with a temperature of 20–25 °C, relative humidity of 40–70%, and a 12 h light/dark cycle. Semi-purified diets were formulated according to the American Institute of Nutrition 1993 (AIN-93) specification, and soybean-derived ingredients were replaced to avoid potential interference from phytoestrogens such as soy isoflavones (Table 1). All rats had free access to food and water throughout the experiment.

Rats in the Sham and OVX groups received sterile physiological saline by oral gavage at 10 mL/kg BW/day. Rats in the OF44 group received OF44 suspension by oral gavage with 5 × 10^8^ CFU/kg BW/day. The intervention lasted for 90 days. Body weight was recorded weekly throughout the intervention period. After the 90-day intervention, all rats were anesthetized by intraperitoneal injection of Zoletil (35 mg/kg) combined with Sumianxin II (7 mg/kg). Following anesthesia, the rats were euthanized by exsanguination. Blood, femurs, colon tissues, and fecal samples were collected immediately thereafter. Blood samples were centrifuged at 4000× *g* for 10 min at 4 °C to separate serum. Serum, femurs, colon tissues, and fecal samples were stored at −80 °C until further analysis. The left femur was sequentially used for Micro-computed tomography (Micro-CT) scanning, biomechanical testing, and bone calcium determination. The right femur was divided into two parts: one part was used for hematoxylin and eosin staining (H&E) and tartrate-resistant acid phosphatase (TRAP) staining, and the remaining part was stored in liquid nitrogen for Western blot analysis. Endpoint analyses used resource-based random subsets of six of the ten rats per group. Subsets were selected using computer-generated random numbers before outcome assessment, independently of measured outcomes. Subsets could differ across assays; microbiota–phenotype correlations used the same six animals per group, matched by individual identifiers. The biological sample size for each assay is reported in the corresponding figure legend. To minimize potential bias, cage placement was performed by a technician blinded to group allocation, while the principal investigator retained the group allocation information. Biological samples were assigned coded identifiers to conceal treatment allocation from investigators performing outcome assessments, including micro-CT analysis, biomechanical testing, ELISA, Western blotting, and histological evaluation (H&E and TRAP staining). For gut microbiota analysis, sample identities were concealed during DNA extraction, sequencing, quality control, and initial data processing, with group information disclosed when required for group-based comparisons. Statistical analyses were performed using coded group identifiers, and the actual treatment identities were revealed after completion of the analyses.

The study evaluated the bone-protective effects of OF44 at 5 × 10^8^ CFU/kg BW/day against the OVX vehicle control, with the Sham group providing the sham-operated reference.

### 2.3. Micro-CT Analysis

Micro-CT scanning was performed using a QX-W400 scanner (Shanghai Qixiang Testing Instruments Co., Ltd., Shanghai, China). The method was adapted from previous reports with slight modifications. The left femur was scanned with the following parameters: resolution: 24 μm, voltage: 70 kV, current: 114 μA, Gaussian filter: σ = 0.8, support = 1, threshold = 330. Bone mineral density (BMD), bone volume/total volume (BV/TV), bone surface area/bone volume (BS/BV), trabecular number (Tb.N), trabecular thickness (Tb.Th), trabecular separation (Tb.Sp), connectivity density (Conn.D), and structure model index (SMI) were analyzed using SCANCO RayTracer 4.3.0.7 modeling software. Trabecular bone analysis was performed on 100 consecutive slices (2.4 mm total height), starting 50 slices (1.2 mm) distal to the femoral growth plate. Three-dimensional (3D) reconstruction was carried out using SCANCO Evaluation software.

### 2.4. Bone Calcium Content

The left femur, which had been subjected to biomechanical testing, was dried in an oven at 105 °C ± 2 °C until a constant weight was achieved to obtain its dry diaphyseal weight. It was then digested, and its calcium content was measured using an inductively coupled plasma mass spectrometer (ICP-MS; model NexION 1000G, PerkinElmer, Waltham, MA, USA).

### 2.5. Measurement of Femoral Biomechanics

Femoral biomechanical properties were measured using a QX-W400 universal testing machine (Shanghai Qixiang Testing Instruments Co., Ltd., Shanghai, China). The left femur was dissected free of soft tissues and subjected to a three-point bending test to determine the maximum load, elastic modulus, maximum bending stress, and maximum strain. The span between the two supports was set at 20 mm. The femur was placed on the supports with the anterior surface facing upward. The distal condyles and the proximal greater trochanter were placed on the two supporting platforms, respectively. The test parameters were: loading speed: 2 mm/min, maximum displacement: 1 mm.

### 2.6. Serum Biochemical Marker Analysis

After the final administration, rats were anesthetized, and blood samples were obtained via the abdominal aorta. Serum levels of bone turnover markers and inflammatory cytokines were quantified using commercial enzyme-linked immunosorbent assay (ELISA) kits (Andygene) according to the manufacturer’s instructions. The analytes included the bone formation markers bone-specific alkaline phosphatase (BALP), osteocalcin (OCN), procollagen type I C-terminal propeptide (PICP), and procollagen type I N-terminal propeptide (PINP); the bone resorption markers tartrate-resistant acid phosphatase 5b (TRACP-5b), C-terminal telopeptide of type I collagen (CTX-I), and N-terminal telopeptide of type I collagen (NTX); the bone regulatory factor osteoprotegerin (OPG); and the inflammatory cytokines tumor necrosis factor-α (TNF-α) and interleukin-10 (IL-10). The supplier, catalog number, and detection range of each ELISA kit are provided in Supplementary Table S1.

### 2.7. Examination of Femoral Histomorphology and Assessment of Osteoclast Activity

The portion of the right femur allocated for histological analysis was fixed in 4% paraformaldehyde for 24 h, decalcified with ethylenediaminetetraacetic acid, and processed for H&E and TRAP staining.

### 2.8. Western Blotting Analysis

The method described by Li et al. was adopted with minor modifications [17]. The femoral head tissue was crushed with bone forceps and transferred to a mortar, where it was ground into a fine powder with the addition of liquid nitrogen. The following proteins were detected: sclerostin (SOST), dickkopf-1 (DKK1), Wnt family member 10b (Wnt10b), β-catenin, glycogen synthase kinase 3β phosphorylated at Tyr216 [p-GSK3β (Tyr216)], runt-related transcription factor 2 (Runx2), alkaline phosphatase (ALP), OPG, receptor activator of nuclear factor κB ligand (RANKL), receptor activator of nuclear factor κB (RANK), nuclear factor of activated T cells cytoplasmic 1 (NFATc1), and tartrate-resistant acid phosphatase (TRAP). Detailed information on the primary and secondary antibodies, including suppliers, catalog numbers, host species, and working dilutions, is provided in Supplementary Table S2.

### 2.9. Gut Microbiota Analysis

Fresh fecal samples were collected from rats at the end of the intervention and immediately stored at −80 °C until DNA extraction. Approximately 0.2–0.5 g of each fecal sample was used for microbial DNA extraction. Total microbial genomic DNA was extracted using the MagBeads FastDNA Kit for Soil (MP Biomedicals, Solon, OH, USA) according to the manufacturer’s instructions. The concentration and purity of the extracted DNA were determined using a NanoDrop NC2000 spectrophotometer (Thermo Fisher Scientific, Waltham, MA, USA), and DNA integrity was assessed by 0.8% agarose gel electrophoresis.

The bacterial 16S rRNA gene V3–V4 region was amplified using the primers 338F (5′-ACTCCTACGGGAGGCAGCA-3′) and 806R (5′-GGACTACHVGGGTWTCTAAT-3′). Sample-specific barcodes were added to distinguish different samples in the same sequencing library. PCR amplification was performed using Q5 High-Fidelity DNA Polymerase. Each 25 μL PCR reaction contained 0.25 μL Q5 high-fidelity DNA polymerase, 5 μL 5× reaction buffer, 5 μL 5× High GC buffer, 2 μL dNTPs, 2 μL template DNA, 1 μL forward primer, 1 μL reverse primer, and 8.75 μL nuclease-free water. The PCR program was as follows: initial denaturation at 98 °C for 5 min; 25 cycles of denaturation at 98 °C for 30 s, annealing at 52 °C for 30 s, and extension at 72 °C for 45 s; followed by a final extension at 72 °C for 5 min. PCR products were examined by 2% agarose gel electrophoresis, purified, and quantified using the Quant-iT PicoGreen dsDNA Assay Kit (Invitrogen, Carlsbad, CA, USA). Purified amplicons were pooled in equimolar amounts for library construction.

Paired-end sequencing was performed by Shanghai Personal Biotechnology Co., Ltd. (Shanghai, China) on the Illumina NovaSeq platform. Project records identify DADA2 quality filtering, denoising, paired-end merging, and chimera removal as the sequence-processing workflow, yielding amplicon sequence variants (ASVs). Taxonomic annotation used Greengenes 13_8. Downstream feature-table processing and rarefaction were documented in QIIME 2 2022.11.1.

For the 18 samples included in the microbiota analysis, the provider reported 67,656–97,066 primer-matched input sequences, 62,811–91,053 filtered sequences, 60,799–88,233 denoised sequences, 49,550–76,031 merged sequences, and 42,264–66,202 non-chimeric sequences per sample. The input counts represent primer-matched sequences rather than untreated raw sequencing reads. Complete sample-level processing counts are provided in the Supplementary Table S3.

After singleton filtering, library sizes ranged from 42,093 to 66,103 sequences. The filtered ASV table was rarefied without replacement to 39,988 sequences per sample for alpha-diversity and Bray–Curtis analyses, retaining all 18 samples. Supplementary Table S4 provides the ASV tables before and after rarefaction. Figure S1 shows the provider-derived Chao1 rarefaction curves for all 18 samples, evaluated at depths up to 37,988 sequences with 10 subsamples per depth.

Alpha-diversity and coverage indices included Chao1, observed species, Shannon diversity, Simpson diversity, Pielou’s evenness, Faith’s phylogenetic diversity, Good’s coverage, Allen’s entropy, and Rao’s quadratic entropy. Bray–Curtis dissimilarities were visualized using principal coordinate analysis (PCoA). The Venn diagram was constructed from the filtered ASV table before rarefaction and was interpreted descriptively. Taxonomic composition was summarized using relative abundances, and differential taxonomic features were evaluated using LEfSe and the additional multiple-testing procedures described in Section 2.10. Six animals per group were included in the microbiota analysis. For microbiota–phenotype correlations, paired microbial relative-abundance and phenotypic observations from the same animals were analyzed across all three groups, giving 18 paired observations in total.

### 2.10. Statistical Analysis

Unless otherwise specified in the figure legends, quantitative data are expressed as mean ± standard deviation (SD). The sample size for each assay is specified in the corresponding figure legend. Before group comparisons, normality and homogeneity of variances were assessed for all quantitative outcomes subjected to one-way ANOVA. No significant deviations from normality were detected. Conventional one-way ANOVA was used for datasets satisfying the homogeneity-of-variance assumption. For datasets with unequal variances, the Brown–Forsythe robust ANOVA was used to compare group means. Statistical analyses of non-microbiota outcomes were conducted using SPSS 26.0 software (IBM, Chicago, IL, USA), and the corresponding graphs were generated using GraphPad Prism 10 software (GraphPad Software, San Diego, CA, USA). For these outcomes, *p* < 0.05 was considered statistically significant. Microbiota statistical reanalyses were performed using Python 3.12.14 with SciPy 1.18.1, scikit-bio 0.7.4, and scikit-posthocs 0.17.1.

For alpha-diversity analyses, overall group differences were evaluated using Kruskal–Wallis tests, with Benjamini–Hochberg (BH) correction across the nine indices. Pairwise differences were evaluated using two-sided, tie-corrected Dunn tests, with BH correction across all 27 index-by-comparison tests.

Differences in beta diversity among the Sham, OVX, and OF44 groups were evaluated using one-way PERMANOVA on Bray–Curtis dissimilarities calculated from the ASV table rarefied to 39,988 sequences per sample. The analysis included 18 animals, with six animals per group. Homogeneity of multivariate dispersion was assessed using PERMDISP based on distances to spatial medians. Both tests used 9999 unrestricted permutations. Pairwise comparisons were subsequently performed, with Benjamini–Hochberg correction applied separately across the three PERMANOVA comparisons and the three PERMDISP comparisons. Omnibus permutation *p* values and pairwise BH-adjusted *p* values are reported separately.

LEfSe used one-against-all comparisons with the Wilcoxon step enabled and a log10 LDA score threshold of 4. Multiple testing was additionally assessed using Kruskal–Wallis statistics reconstructed from the LEfSe input, with Benjamini–Hochberg (BH) correction across all 167 hierarchical taxonomic entries. Features included in the differential taxonomic visualization met both criteria: an original LEfSe LDA score ≥ 4 and a BH-adjusted Kruskal–Wallis *p* value < 0.05. Supplementary Table S7 reports test statistics, unadjusted and BH-adjusted *p* values, group mean relative abundances, and the original LEfSe LDA effect-size scores where available.

Spearman correlations were calculated using the 18 paired animal observations pooled across the three groups. Two-sided *p* values were estimated from 99,999 random permutations of phenotype labels across the 18 animals, using the absolute Spearman correlation coefficient as the test statistic and the plus-one correction. Benjamini–Hochberg correction was applied across all 108 combinations of six bacterial families and 18 measured phenotypic variables. For these correlation analyses, statistical significance was defined as a BH-adjusted *p* value < 0.05. Complete Spearman correlation coefficients, unadjusted permutation *p* values, and BH-adjusted *p* values are provided in the Supplementary Table S8.

## 3. Results and Discussion

### 3.1. OF44 Improved Femur Microstructure in OVX Rats

**Figure 1:**
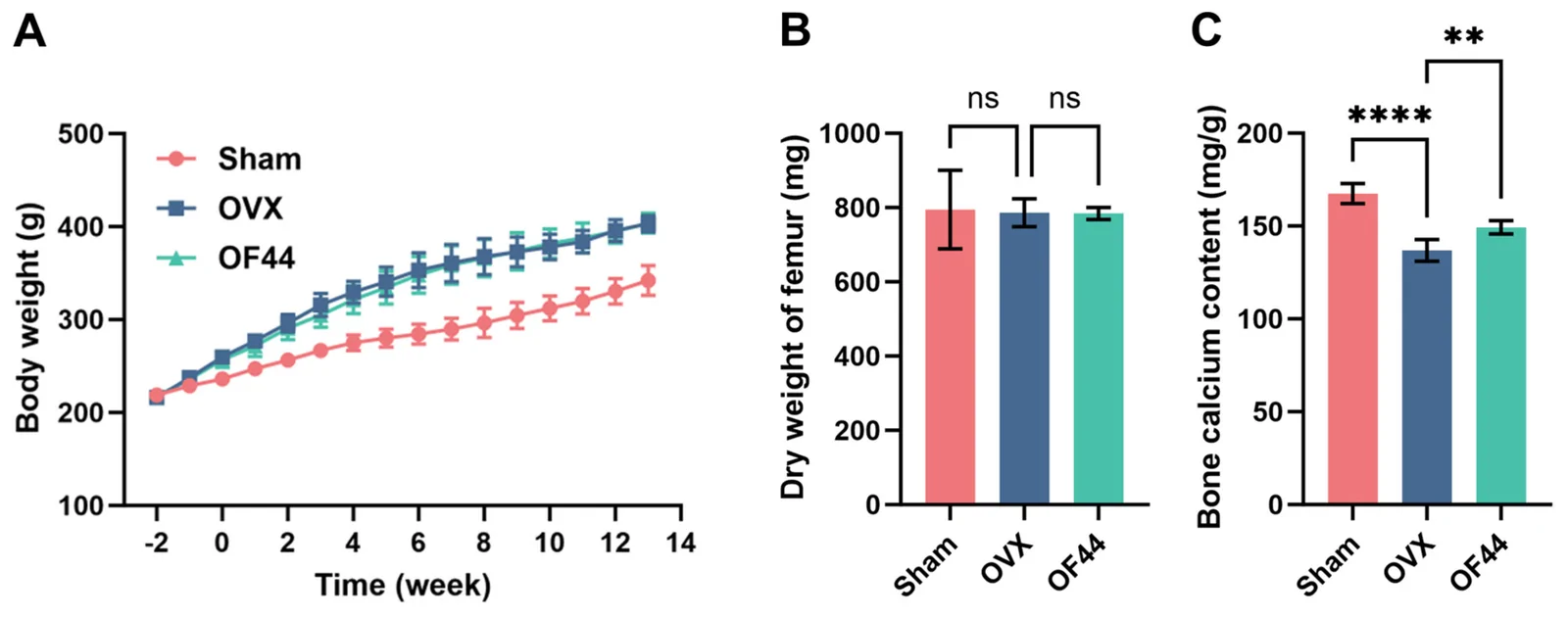
(**A**) Changes in body weight of experimental animals over the course of the experimental period. (**B**) Differences in femoral shaft weight among groups. (**C**) Bone calcium content. Data are presented as mean ± standard deviation (*n* = 6). ** *p* < 0.01, **** *p* < 0.0001; ns, not significant.

Bone calcium content was significantly lower in the OVX group than in the Sham group (Figure 1C), supporting the skeletal phenotype of the model. Femoral dry weight did not differ significantly among groups (Figure 1B); cortical and trabecular bone have different turnover rates [18,19]. Body weight was significantly higher in both the OVX and OF44 groups than in the Sham group during the intervention (Figure 1A), consistent with estrogen deficiency-related changes in metabolic regulation [20]. OF44-associated improvements in bone microarchitecture occurred without a significant reduction in body weight or change in femoral shaft weight [21].

**Figure 2:**
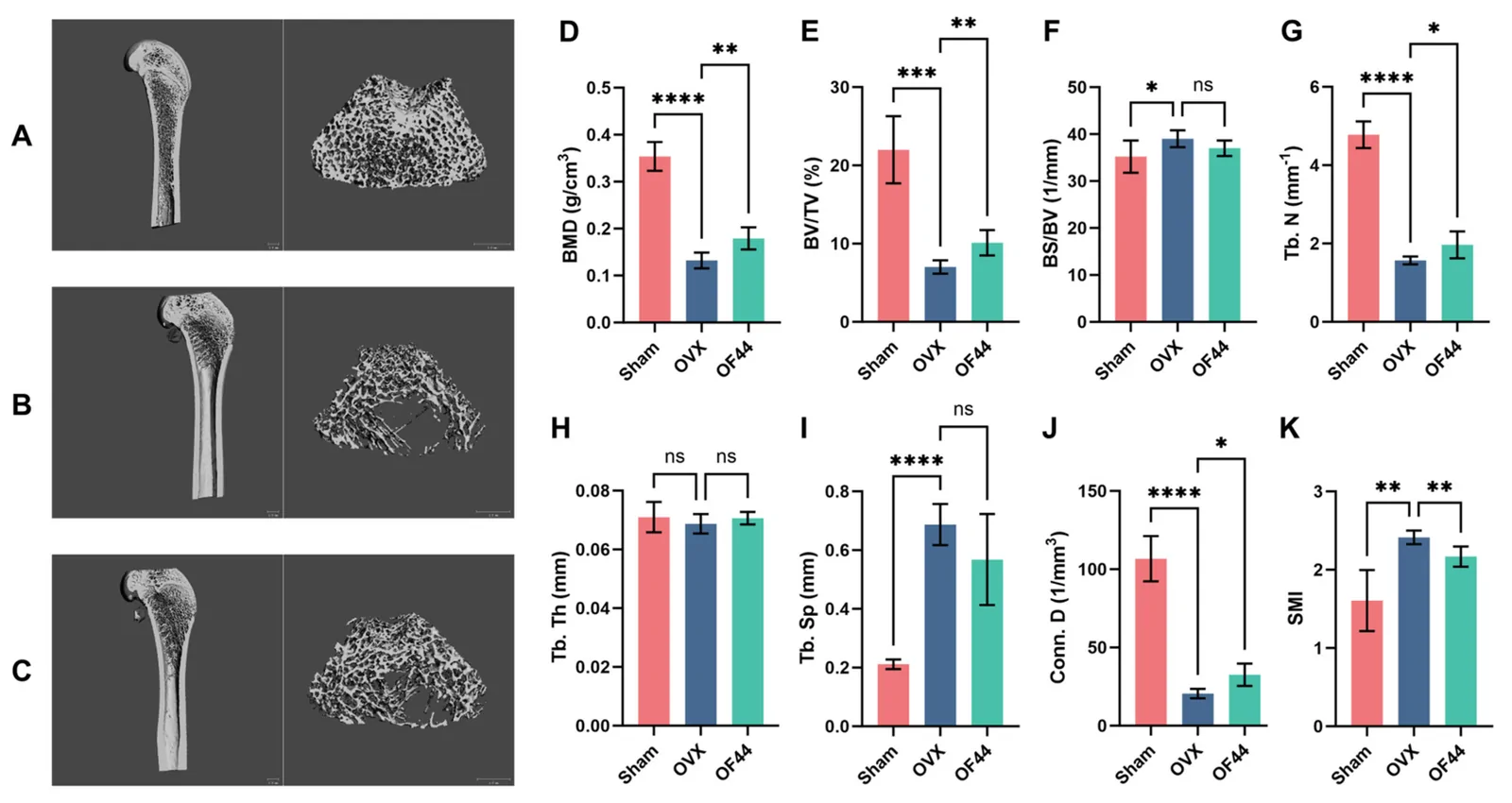
OF44 ameliorated OP in ovariectomized rats. The distal femoral metaphyseal region of rats was analyzed using Micro-CT. Representative Micro-CT images are shown for the (**A**) Sham group, (**B**) OVX group, and (**C**) OF44 group. The scale bar in panels (**A**–**C**) represents 1.0 mm. (**D**–**K**) BMD, BV/TV, BS/BV, Tb.N, Tb.Th, Tb.Sp, Conn.D, and SMI, respectively. Data are presented as mean ± standard deviation (*n* = 6). * *p* < 0.05, ** *p* < 0.01, *** *p* < 0.001, **** *p* < 0.0001; ns, not significant.

OF44 improved distal femoral trabecular architecture in OVX rats. Micro-CT showed better-preserved trabecular structure after OF44 treatment (Figure 2A–C), together with significantly higher BMD, BV/TV, Tb.N, and Conn.D and lower SMI than in OVX controls (Figure 2D–K). The lower SMI was consistent with a shift toward more plate-like trabecular architecture [22]. The OVX–OF44 differences in Tb.Sp, BS/BV, and Tb.Th were not statistically significant. Together, the density, bone volume, connectivity, and architectural measurements demonstrate preservation of key trabecular properties after OF44 administration.

Estrogen deficiency disrupts the balance between bone resorption and formation [23]. The structural improvements after OF44 treatment support its bone-protective effect in this model [24].

### 3.2. OF44 Improved the Biomechanical Properties of the Femur in OVX Rats

**Figure 3:**
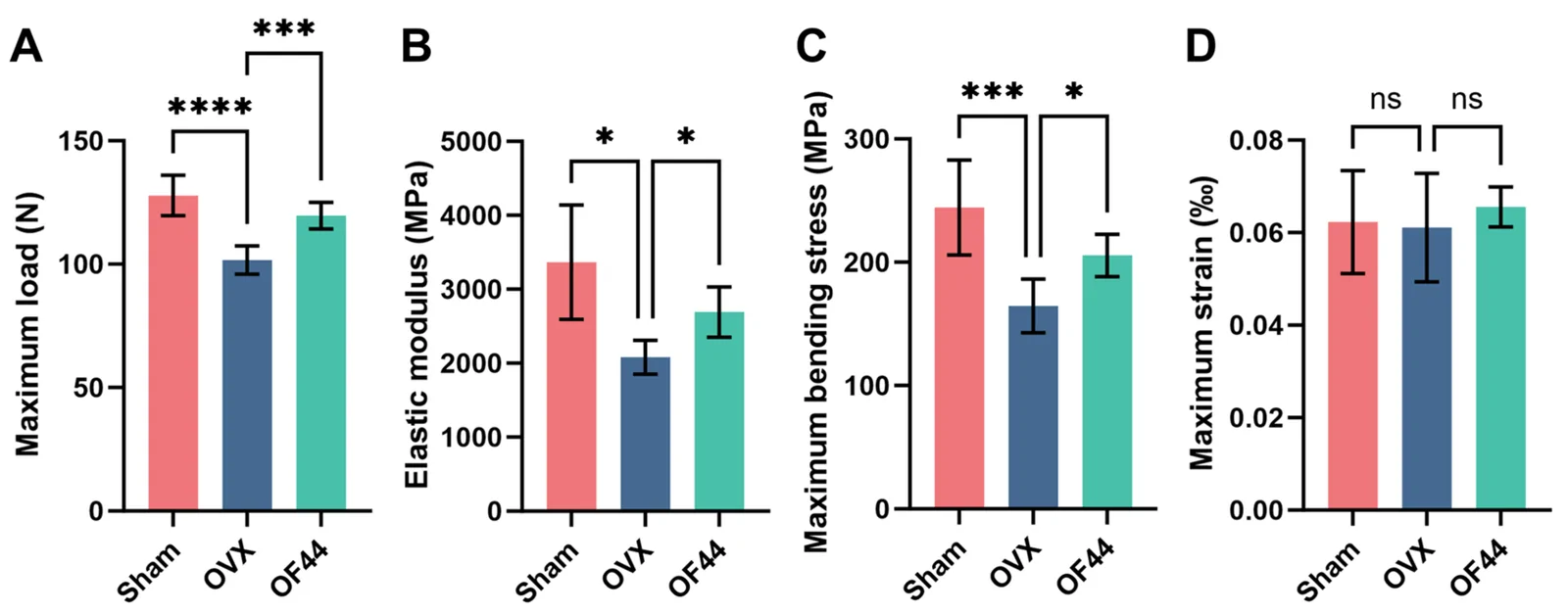
Effects of OF44 on femoral biomechanical properties in rats. (**A**) Maximum load, (**B**) elastic modulus, (**C**) maximum bending stress, and (**D**) maximum strain of the femur in Sham, OVX, and OF44 groups. Data are presented as mean ± standard deviation (*n* = 6). * *p* < 0.05, *** *p* < 0.001, **** *p* < 0.0001; ns, not significant.

OF44 significantly increased femoral maximum load, elastic modulus, and maximum bending stress compared with OVX controls (*p* < 0.05; Figure 3A–D). OVX rats had significantly lower values for these three endpoints than Sham rats, consistent with impaired whole-bone strength under estrogen deficiency [25]. The improvements after OF44 treatment extend the micro-CT findings to load-bearing capacity and mechanical stiffness and align with reports of improved bone quality in estrogen-deficient models [26]. Maximum strain, a measure of deformation rather than load-bearing capacity, did not differ significantly among groups [27].

### 3.3. OF44 Improved the Serum Bone Turnover Profile in OVX Rats

**Figure 4:**
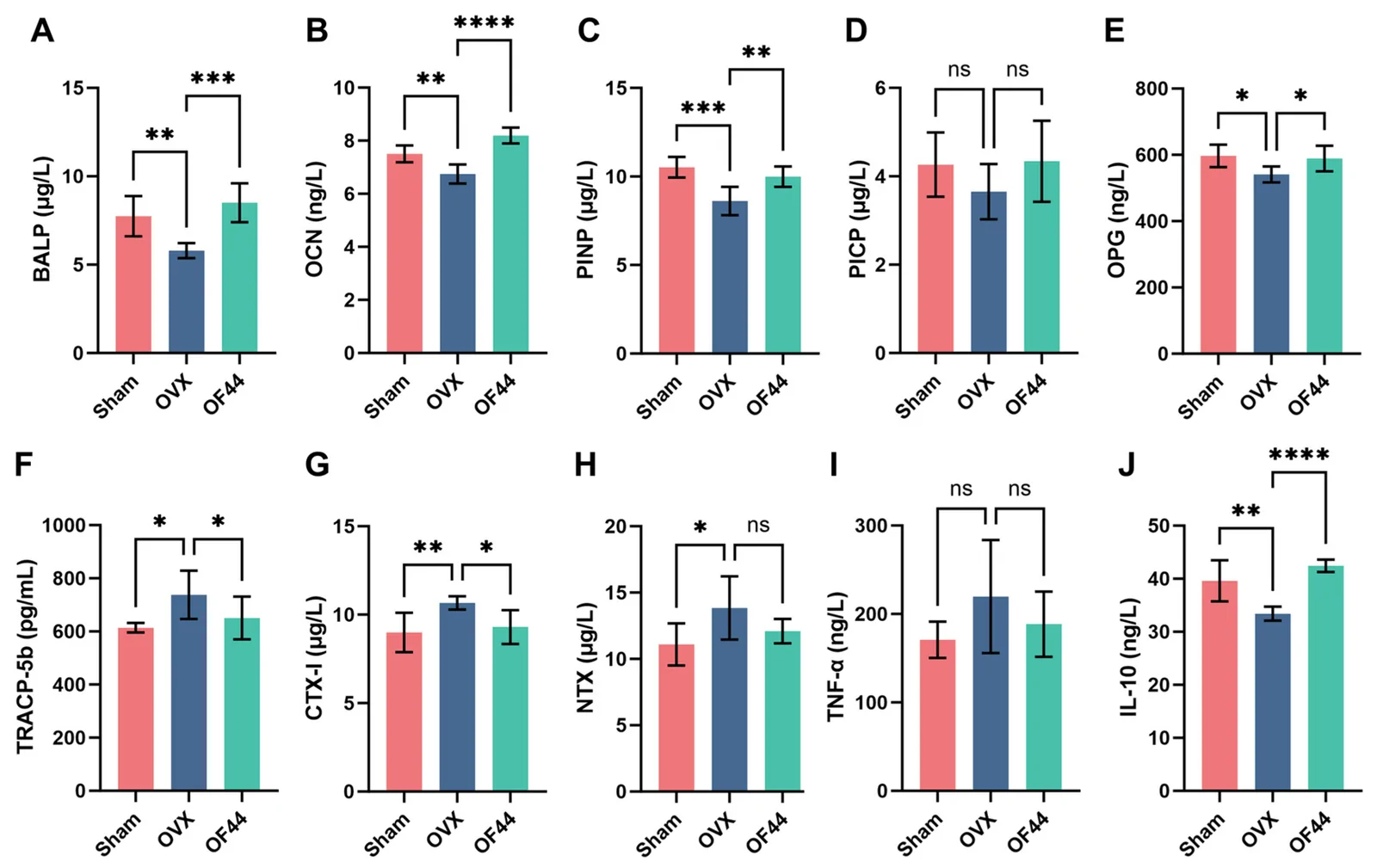
Effects of OF44 on serum bone turnover markers and cytokine levels in ovariectomized rats. (**A**) BALP, (**B**) OCN, (**C**) PINP, (**D**) PICP, (**E**) OPG, (**F**) TRACP-5b, (**G**) CTX-I, (**H**) NTX, (**I**) TNF-α, and (**J**) IL-10 levels in the serum of Sham, OVX, and OF44 groups. Data are presented as mean ± standard deviation (*n* = 6). * *p* < 0.05, ** *p* < 0.01, *** *p* < 0.001, **** *p* < 0.0001; ns, not significant.

OF44 significantly increased serum BALP, OCN, PINP, and OPG relative to OVX controls (Figure 4A–E). OVX rats had significantly lower values for these four markers than Sham rats, while PICP did not differ significantly among groups. The increases in formation-related markers and OPG characterize a favorable serum bone turnover profile [28].

OF44 significantly reduced serum TRACP-5b and CTX-I relative to OVX controls (Figure 4F–H). OVX rats had significantly higher TRACP-5b, CTX-I, and NTX than Sham rats, consistent with increased bone resorption [29]. NTX was numerically lower in OF44-treated rats than in OVX controls, but the difference was not statistically significant. Together with the increases in BALP, OCN, PINP, and OPG, these findings show an OF44-associated shift toward a more favorable serum bone turnover profile.

### 3.4. OF44 Increased Serum IL-10 in OVX Rats

OF44 significantly increased serum IL-10 compared with OVX controls, in which IL-10 was significantly lower than in Sham rats (Figure 4J). Serum TNF-α did not differ significantly among the three groups (Figure 4I).

IL-10 provides a biological context for the accompanying changes in bone-related outcomes after OF44 administration. Estrogen deficiency can alter immune regulation and promote osteoclastogenesis [30], while IL-10 has been reported to suppress osteoclast differentiation [31]. In this study, increased serum IL-10 accompanied lower osteoclast-related markers and fewer TRAP-positive osteoclasts. This combination supports an association between the IL-10 response and the bone-protective phenotype, consistent with previous probiotic studies in estrogen-deficient models [9,32].

### 3.5. Preserved Trabecular Histomorphology and Reduced Osteoclast Numbers in OVX Rats

**Figure 5:**
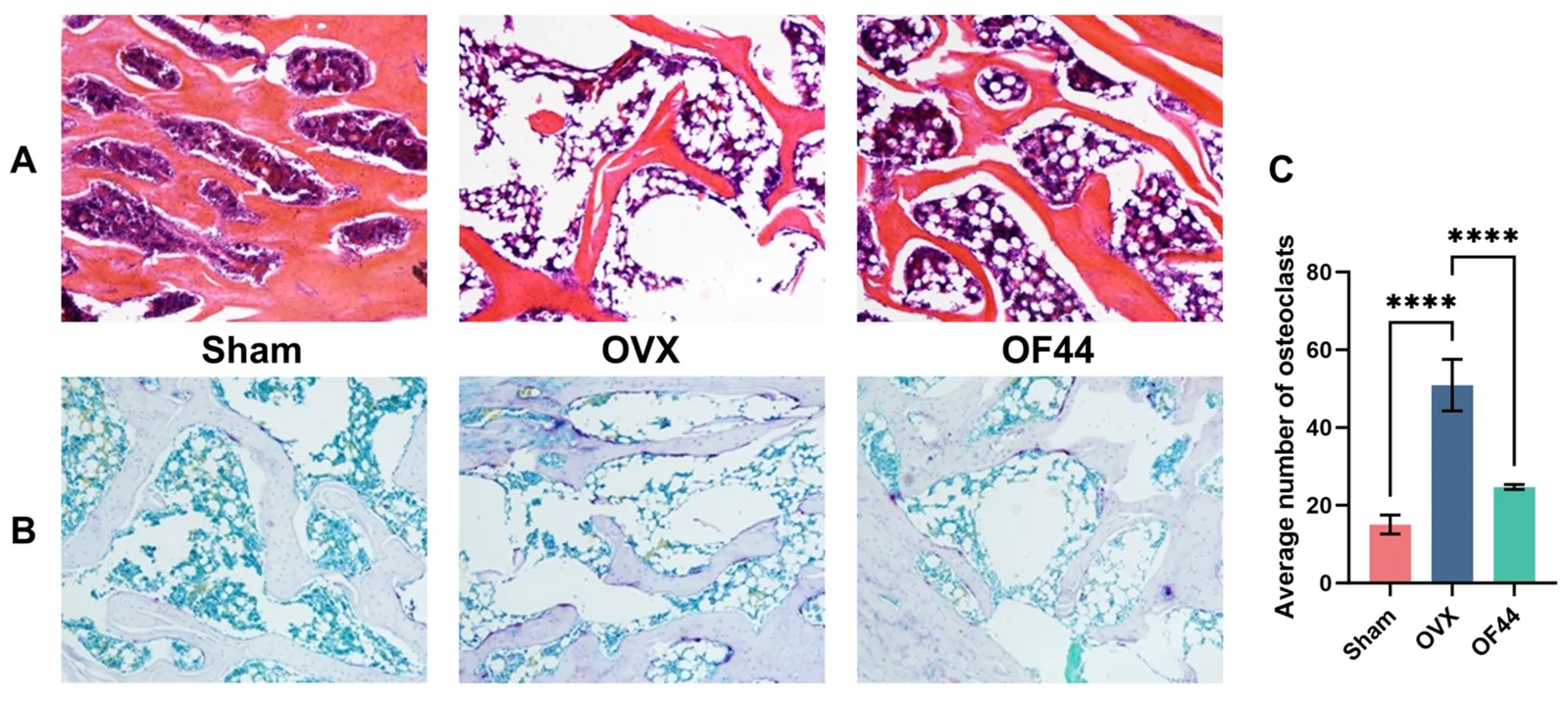
Effects of OF44 on femoral microstructure and osteoclast activity in ovariectomized rats. (**A**) H&E staining of femoral tissue sections showing trabecular morphology. (**B**) TRAP staining for osteoclasts. The images in panels (**A**) and (**B**) were captured at ×100 magnification. (**C**) Quantitative analysis of osteoclast number. Data are presented as mean ± standard deviation (*n* = 6). **** *p* < 0.0001.

OF44 preserved trabecular morphology and significantly reduced TRAP-positive osteoclast numbers in OVX rats (Figure 5). H&E images showed better-organized trabeculae and qualitatively less marrow adiposity in OF44-treated rats than in OVX controls, whose trabeculae showed severe deterioration and disordered arrangement (Figure 5A). OVX rats also showed more TRAP-positive osteoclasts than Sham rats, while OF44 treatment significantly reduced osteoclast counts (Figure 5B,C) [33].

The reduction in TRAP-positive osteoclasts provides a cellular counterpart to the lower serum resorption markers and preserved trabecular microarchitecture. Marrow adipocytes can produce RANKL and adipokines involved in osteoclast differentiation [34], placing the observed adiposity pattern in a bone-remodeling context. Together, these histological and biochemical findings connect trabecular preservation with fewer osteoclasts after OF44 treatment.

### 3.6. OF44 Modulated Bone-Remodeling-Related Protein Expression in OVX Rats

**Figure 6:**
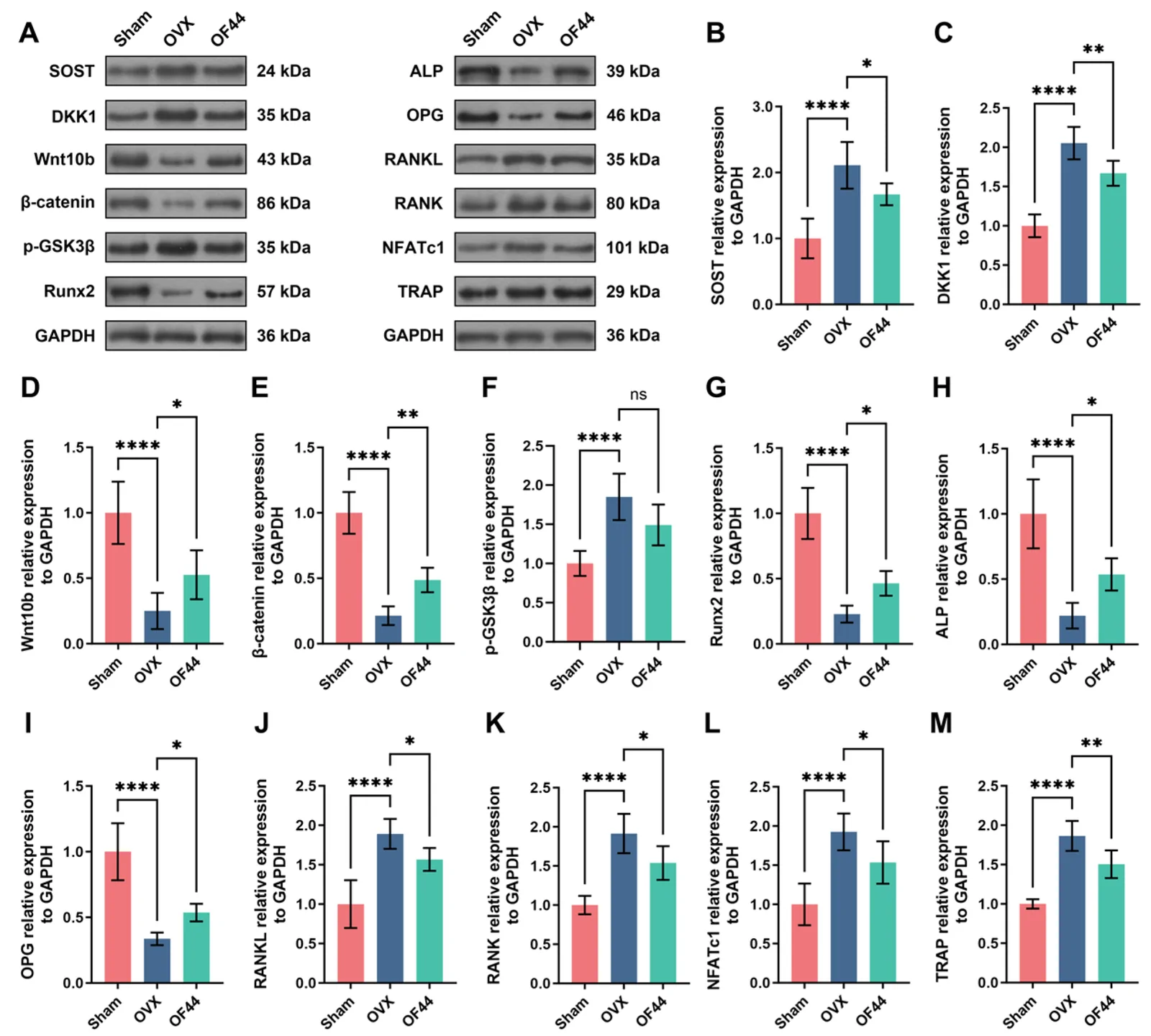
Effects of OF44 on Wnt/β-catenin- and RANKL/RANK/OPG-related protein expression in the femur of ovariectomized rats. (**A**) Representative Western blot images of the indicated proteins. (**B**–**M**) Quantitative analysis of relative protein expression normalized to GAPDH. Data are presented as mean ± standard deviation (*n* = 6). * *p* < 0.05, ** *p* < 0.01, **** *p* < 0.0001; ns, not significant.

The Wnt/β-catenin and RANKL/RANK/OPG signaling systems participate in the regulation of bone remodeling [35,36,37]. Western blot analysis showed that, compared with the Sham group, the OVX group had higher SOST, DKK1, RANKL, and RANK protein expression and lower Wnt10b, β-catenin, and OPG protein expression (Figure 6).

Compared with the OVX group, OF44-treated rats showed increased expression of Wnt10b, β-catenin, Runx2, ALP, and OPG, together with decreased expression of SOST, DKK1, RANKL, RANK, NFATc1, and TRAP (Figure 6). The GAPDH-normalized p-GSK3β (Tyr216) signal did not differ significantly between the OVX and OF44 groups; the β-catenin increase is therefore interpreted in relation to the broader protein-expression profile. This protein profile accompanied the improvements in bone structure and mechanical performance and the reduction in TRAP-positive osteoclasts.

### 3.7. OF44 Altered Gut Microbial Diversity and Composition in OVX Rats

#### 3.7.1. Gut Microbial Diversity and Community Composition

**Figure 7:**
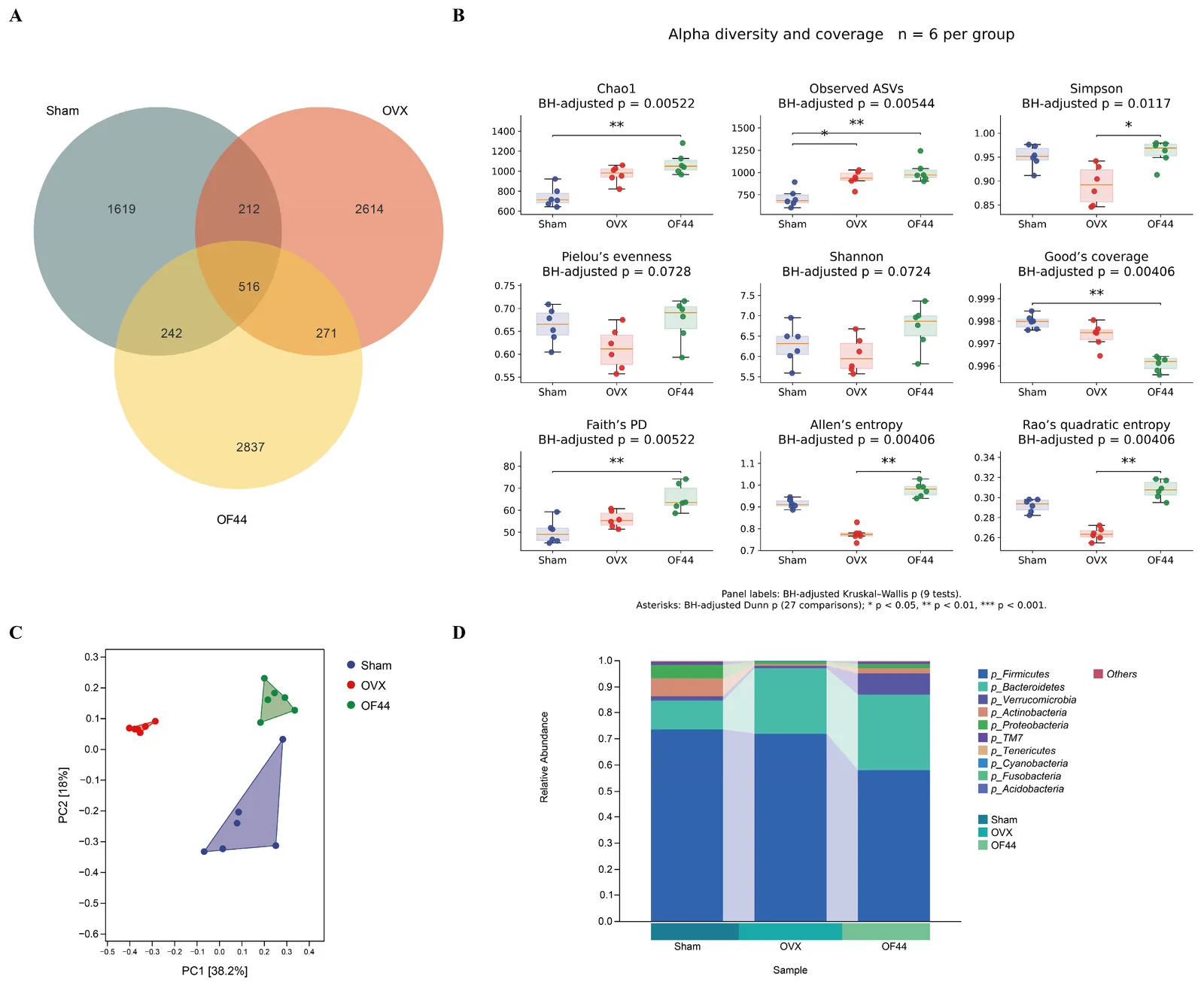
Gut microbiota composition and diversity in Sham, OVX, and OF44 groups. (**A**) Venn diagram showing shared and group-specific ASVs based on the filtered table before rarefaction. The blue-gray, salmon, and pale-yellow circles represent the Sham, OVX, and OF44 groups, respectively. Overlapping regions indicate shared ASVs among the groups. (**B**) Alpha-diversity and coverage indices calculated after rarefaction to 39,988 sequences per sample. Boxes show the median and interquartile range; whiskers extend to the most extreme observations within 1.5 times the interquartile range, and points represent individual animals. Panel labels report Kruskal–Wallis *p* values after BH correction across nine indices. Pairwise comparisons used Dunn tests with BH correction across all 27 comparisons. One and two asterisks indicate BH-adjusted Dunn *p* values < 0.05 and < 0.01, respectively. (**C**) PCoA based on Bray–Curtis dissimilarities. Overall group differences were evaluated using PERMANOVA (pseudo-*F*_2_,_15_ = 7.7597, *R*^2^ = 0.5085, *p* = 0.0001). PERMDISP detected heterogeneous group dispersion (*F* = 2.0244, *p* = 0.0202). Both tests used 9999 permutations. Complete alpha-diversity and beta-diversity statistics are provided in Supplementary Tables S5 and S6, respectively; Chao1 rarefaction curves are shown in Figure S1. (**D**) Mean relative abundances of the top 10 bacterial phyla in each group. Six animals were analyzed per group.

At the group level, the filtered ASV table before rarefaction contained 2589, 3613, and 3866 ASVs across the six samples in the Sham, OVX, and OF44 groups, respectively (Figure 7A).

Compared with the OVX group, the OF44 group had higher Simpson diversity, Allen’s entropy, and Rao’s quadratic entropy after BH correction across all 27 Dunn comparisons (BH-adjusted *p* = 0.0135, 0.00257, and 0.00257, respectively; Figure 7B). Across the three groups, seven of the nine alpha-diversity and coverage indices showed overall differences after BH correction; Shannon diversity and Pielou’s evenness did not reach the adjusted significance threshold.

PCoA based on Bray–Curtis dissimilarities showed group-related differences, with the first two axes explaining 38.2% and 18.0% of the variation, respectively (Figure 7C). Formal PERMANOVA detected an overall difference among the three groups (pseudo-*F*_2_,_15_ = 7.7597, *R*^2^ = 0.5085, *p* = 0.0001; 9999 permutations). All three pairwise PERMANOVA comparisons remained significant after BH correction (Sham versus OVX, Sham versus OF44, and OVX versus OF44; BH-adjusted *p* = 0.0021 for each comparison).

PERMDISP detected heterogeneous group dispersion (*F* = 2.0244, *p* = 0.0202), including an OVX–OF44 difference after pairwise BH correction (BH-adjusted *p* = 0.0063). Unequal dispersion may therefore contribute to the PERMANOVA group differences. Complete omnibus and pairwise results are provided in Supplementary Table S6.

Relative to OVX controls, the OF44 group had a lower mean proportion of *Firmicutes* (57.87% versus 71.91%) and higher mean proportions of *Bacteroidetes* (29.15% versus 25.17%) and *Verrucomicrobia* (8.16% versus 0.93%). The corresponding Sham values were 73.61%, 11.07%, and 1.71%, respectively (Figure 7D).

#### 3.7.2. Characteristic Taxa and Microbiota–Phenotype Associations

**Figure 8:**
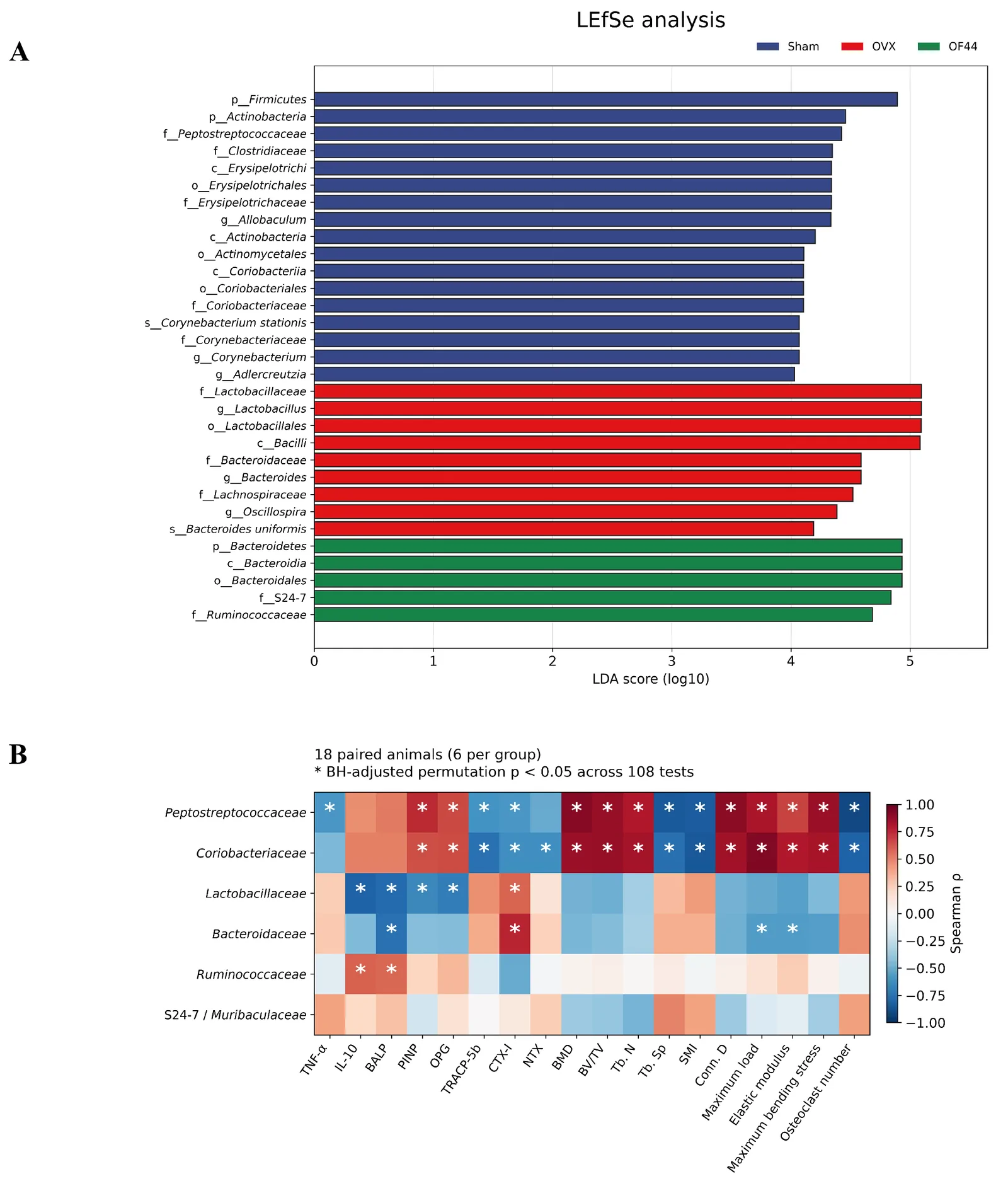
Differential taxonomic entries and exploratory microbiota–phenotype correlations. (**A**) LEfSe effect sizes for 31 named taxonomic entries. Bar length represents the original LDA score on the log10 scale. All displayed entries had LDA scores ≥ 4 and BH-adjusted Kruskal–Wallis *p* values < 0.05, with BH adjustment applied across all 167 tested taxonomic entries. Sham-, OVX-, and OF44-associated entries are shown in blue, red, and green, respectively. Complete taxonomic statistics, including 12 additional Unclassified entries, are provided in Supplementary Table S7. (**B**) Spearman correlations between six bacterial families and 18 measured phenotypic variables, using 18 paired animal observations pooled across groups (six animals per group). Red and blue indicate positive and negative correlations, respectively; color intensity represents Spearman’s ρ. An asterisk denotes a BH-adjusted two-sided permutation *p* value < 0.05, with adjustment applied across all 108 comparisons. Complete correlation results are provided in Supplementary Table S8.

LEfSe identified an OF44-associated taxonomic profile that included S24-7 (LDA score = 4.84; BH-adjusted *p* = 0.00818) and Ruminococcaceae (LDA score = 4.68; BH-adjusted *p* = 0.0429). Across the full analysis, 43 hierarchical taxonomic entries had LDA scores ≥ 4 and BH-adjusted Kruskal–Wallis *p* values < 0.05, with BH adjustment across all 167 tested entries. Figure 8A displays 31 named entries, and the remaining 12 Unclassified entries, individual LDA scores, and group mean relative abundances are provided in Supplementary Table S7. These entries describe a hierarchical taxonomic profile because multiple ranks within the same lineage are represented. OVX-associated entries included Lactobacillaceae, *Lactobacillus*, Lactobacillales, Bacilli, Bacteroidaceae, *Bacteroides*, *Oscillospira*, Lachnospiraceae, and *Bacteroides uniformis*. The OVX-associated *Lactobacillus* signal is a genus-level observation, and the functions of individual species and strains within this genus differ [38,39].

The reported metabolic potential of members of Ruminococcaceae and S24-7/Muribaculaceae [40,41,42] provides a rationale for investigating the functional relevance of their OF44-associated enrichment.

Of the 108 bacterial family–phenotype associations tested in 18 paired animals, 41 met the significance criterion after two-sided permutation testing and BH correction (BH-adjusted *p* < 0.05; Figure 8B). Peptostreptococcaceae and Coriobacteriaceae showed positive associations with structural and mechanical endpoints, including BMD, BV/TV, maximum load, and elastic modulus, and negative associations with TRACP-5b, CTX-I, and osteoclast number. Ruminococcaceae, an OF44-associated family, was positively correlated with IL-10 (ρ = 0.5893, BH-adjusted *p* = 0.0367) and BALP (ρ = 0.5760, BH-adjusted *p* = 0.0397). Complete coefficients and *p* values for all six families and 18 measured phenotypes are provided in Supplementary Table S8.

The study establishes the skeletal response to OF44 at 5 × 10^8^ CFU/kg BW/day using Sham and OVX comparators. The design evaluated one dose without a pharmacological control; endpoint analyses used six animals per group without an a priori power calculation. Microbiota–phenotype correlations pooled 18 animals and are exploratory. False-positive associations remain possible after BH correction, and group differences, relative-abundance compositionality, and cage-related dependence can influence interpretation; permutations were unrestricted. Western blots and 16S sequencing characterize protein abundance and taxonomic composition; pathway activation and microbial mediation require direct functional testing. GSK3β phosphorylation relative to total GSK3β, kinase activity, β-catenin nuclear localization, TCF/LEF-dependent and canonical Wnt target transcription, intestinal barrier integrity, and SCFA concentrations were not assessed. These findings prioritize dose-ranging studies, pathway-specific functional assays, and targeted microbial interventions to test candidate mechanisms accompanying the skeletal benefits.

## 4. Conclusions

OF44 attenuated ovariectomy-induced bone loss in rats, increasing femoral bone mineral density by 35.5%, trabecular bone volume fraction by 44.1%, and maximum load by 17.7% relative to OVX controls. These structural and mechanical benefits were accompanied by favorable bone turnover markers, fewer TRAP-positive osteoclasts, and increased circulating IL-10. Wnt/β-catenin- and RANKL/RANK/OPG-related protein profiles and gut microbial composition provided associated biological context, including OF44-associated enrichment of *Ruminococcaceae* and S24-7/*Muribaculaceae*. The convergent skeletal outcomes provide strain-specific preclinical evidence for OF44 as a probiotic candidate for bone health under estrogen-deficient conditions.

## Figures and Tables

**Table 1:** Reference feed formulation information. Notes: ^a^: Provided (per kg of diet): MnSO_4_, 110 mg; CuSO_4_, 0.8 mg; FeSO_4_, 1.2 mg; KI, 18 mg; ZnSO_4_, 2960 mg; CaHPO_4_, 2890 mg; MgSO_4_, 1.25 × 10^4^ mg; ^b^: Provided (per kg of diet): Vitamin A, 1.4 × 10^4^ IU; Vitamin D, 1500 IU; Vitamin E, 120 mg; Vitamin K, 3 mg; Vitamin B_1_, 12 mg; Vitamin B_2_, 20 mg; Vitamin B_6_, 12 mg; Vitamin B_12_, 0.03 mg; Niacin, 60 mg; Pantothenic acid, 24 mg; Folic acid, 6 mg; Biotin, 0.54 mg.

Ingredients	Content (%)
Casein	23
DL-methionine	0.3
Cornstarch	32
Sucrose	30
Fiber	5
Corn oil	5
Mixed Mineral Salts ^a^	3.5
Multivitamin ^b^	1
Bitartrate Choline	0.2

## Data Availability

The original contributions presented in this study are included in the article/Supplementary Materials. Further inquiries can be directed to the corresponding author.

## Supplementary Materials

The following supporting information can be downloaded at: https://www.mdpi.com/article/10.3390/foods15213902/s1. Supplementary Methods: Details of sequencing processing, rarefaction, diversity analyses, LEfSe-related analyses, exploratory Spearman correlations, and statistical reporting. Figure S1: Chao1 rarefaction curves for the 18 analyzed samples from the Sham, OVX, and OF44 groups. Table S1: Details of the ELISA kits used for the determination of serum bone turnover markers and inflammatory cytokines in rats. Table S2: Details of the primary and secondary antibodies used for Western blot analysis of femoral proteins in rats. Table S3: Sample information and sequence counts at successive processing stages of 16S rRNA gene sequencing. Table S4: Amplicon sequence variant (ASV) count matrices before singleton filtering, after filtering but before rarefaction, and after rarefaction to 39,988 sequences per sample, with sample metadata and depth checks. Table S5: Sample-level alpha-diversity indices and Kruskal–Wallis and Dunn test results with Benjamini–Hochberg-adjusted *p* values. Table S6: Overall and pairwise PERMANOVA and PERMDISP results, the Bray–Curtis dissimilarity matrix, and principal coordinates analysis coordinates. Table S7: Hierarchical taxonomic entries used in LEfSe analysis, including available provider LDA scores, additional Kruskal–Wallis statistics, Benjamini–Hochberg-adjusted *p* values, and group mean relative abundances. Table S8: Exploratory Spearman correlations between six bacterial families and 18 measured phenotypes, including correlation coefficients, permutation *p* values, Benjamini–Hochberg-adjusted *p* values, paired observations, and variable definitions.
