# Supplementary material for: Lacticaseibacillus rhamnosus OF44-15Ph10T Attenuates Bone Loss in Ovariectomized Rats
Source: Foods. 2026 Oct 30;15(21):3902. doi: 10.3390/foods15213902 (PMC13762766; doi:10.3390/foods15213902)
Supplement: Supplementary file 1 [file foods-15-03902-s001.zip › Supplementary Methods and Figure S1.pdf]

Supplementary materials

Manuscript foods-4601508 | Supplementary methods and Figure S1

Guide to the accompanying data

These materials describe the 18 biological samples used for the microbiota analyses: Sham, OVX, and OF44 (6 animals per group). Tables S1 and S2 are supplied separately. The additional tables are organized as follows.

Item	Contents	Location
Table S3	Sample information and sequencing processing counts	Excel workbook
Table S4	ASV count matrices at three processing stages, sample mapping, and depth checks	Separate TSV archive
Table S5	Individual alpha diversity indices; 9 overall tests and 27 pairwise comparisons	Excel workbook
Table S6	PERMANOVA and PERMDISP; distance matrix and PCoA coordinates	Excel workbook
Table S7	167 hierarchical LEfSe entries and additional Kruskal-Wallis BH analysis	Excel workbook
Table S8	108 exploratory Spearman correlations and the 18 paired observations	Excel workbook
Figure S1	Chao1 rarefaction curves for the 18 analyzed samples	Page 4; values in workbook

Data files. Supplementary_Tables_S3_S5_S8_revised_20261026.xlsx contains Table S3 and Tables S5-S8. Supplementary_Table_S4_ASV_counts.zip contains the complete count matrices as tab-separated text files, together with an English README.

S1. Sequencing processing and count-table provenance

Table S3 preserves the provider-reported Input, Filtered, Denoised, Merged, Non-chimeric, and Non-singleton counts. *Input* is the count after matching both primer sequences; it is not an unprocessed raw read count. Raw sequencing counts and Q20/Q30 summaries were not available in the retrieved records and are marked NR (not reported).

DADA2 was identified as the denoising method. Its original version and the executed trimming, truncation, maxEE, truncQ, paired-end merging, and chimera-removal parameters could not be verified from the available records. Default settings have not been substituted for missing execution parameters.

Processing-report counts and ASV matrix sums are retained separately. For J_40 and L_2, the two unrarefied matrices contain 2 and 3 fewer sequences, respectively, than the corresponding provider QC counts. The source of these small discrepancies is unresolved. No count was altered to force agreement. Table S4 includes stage-specific matrix sums and numbers of nonzero ASVs. Rows with zero counts across the selected 18 samples are retained; the number of exported feature rows is therefore not the number of ASVs detected in these samples.

S2. Rarefaction and alpha diversity

After singleton filtering, samples were rarefied without replacement to 39,988 sequences per sample. This was the selected analysis depth; the minimum available filtered library depth was 42,093. All 18 rarefied libraries sum to 39,988. The unrarefied and rarefied matrices are supplied in Table S4.

Figure S1 uses the saved provider Chao1 rarefaction values for these same 18 samples. Each plotted value is the mean of 10 rarefaction iterations at the indicated depth. The available depth grid ranges from 1 to 37,988 sequences. No curve values beyond this grid were extrapolated, including at the 39,988-sequence analysis depth. The numerical values underlying Figure S1 are included in the workbook.

Individual values for all nine alpha diversity indices are provided in Table S5a. Group differences were evaluated using tie-corrected Kruskal-Wallis tests. Benjamini-Hochberg (BH) adjustment was applied across the nine overall tests (Table S5b). Two-sided, tie-corrected Dunn comparisons were calculated for all three group pairs for each index. A separate BH adjustment was applied jointly across all 27 pairwise comparisons (Table S5c). Signed Dunn Z statistics are defined as group 1 minus group 2; both group mean ranks are provided. These BH-adjusted values should not be interchanged with the within-index Holm-adjusted three-comparison values from the provider.

S3. Beta diversity and permutation tests

The 18-sample Bray-Curtis distance matrix and the first three provider PCoA coordinates are supplied in Tables S6b and S6c. Group differences were evaluated by PERMANOVA for the overall three-group comparison and each of the three group pairs. PERMDISP was used to evaluate differences in multivariate dispersion using distances to the group spatial medians. PERMDISP used eigen-decomposition (eigh) and all coordinate dimensions, rather than a two-axis PCoA projection.

Each test used 9,999 unrestricted label permutations. The overall tests used seed 20261020. Pairwise tests used seeds 20261021 (Sham versus OVX), 20261022 (Sham versus OF44), and 20261023 (OVX versus OF44). BH adjustment was applied separately to the three pairwise PERMANOVA p values and the three pairwise PERMDISP p values. Overall p values were not included in either pairwise adjustment family. Table S6a reports sample sizes, statistics, degrees of freedom, numerical p values, permutation counts, seeds, and PERMANOVA R^2.

The overall PERMDISP p value was 0.0202, and the OVX versus OF44 pairwise dispersion comparison had BH-adjusted $p = 0.0063$. PERMANOVA findings therefore cannot be attributed solely to differences in group centroids. Cage identifiers were unavailable; permutations were not restricted by cage, and potential cage-related dependence was not modeled.

S4. LEfSe and additional multiple-comparison analysis

The saved provider LEfSe input contained 52 terminal taxonomic paths. Descendant abundances were aggregated to obtain 167 hierarchical entries across the input taxonomic levels. Kruskal-Wallis H statistics and p values were calculated for all 167 entries, and BH adjustment was applied across this complete set. These are related, nested taxonomic features, rather than 167 independent biological lineages.

S4. LEfSe analysis (continued)

All 111 Kruskal-Wallis p values reported in the provider output were reproduced to an absolute difference below 2×10^{-16}; the remaining 56 were calculated from the same saved input. In total, 111 of 167 entries had BH-adjusted $p < 0.05$. All 43 entries in the provider output at LDA ≥ 4 met this additional criterion. Figure 8A shows the 31 entries with named terminal labels; the other 12 entries with Unclassified terminal labels remain available in Table S7.

The original provider LDA scores were retained and were not recalculated. NA indicates a score or original p value not reported in the thresholded provider output, rather than a value of zero. The full original LEfSe Wilcoxon and LDA bootstrap procedure was not independently reproduced. The added BH procedure concerns the Kruskal-Wallis stage and does not imply that all stages of the original LEfSe workflow were revalidated.

The column *Hierarchy level in provider input* refers to the position within the saved taxonomic path. Unclassified labels retain their lowest resolved ancestor and do not establish identification at the displayed hierarchy level. Informal codes such as S24-7, F16, and SMB53 are retained as source labels. Table S7 includes the full taxonomic path, group mean proportions, available original statistics, added test results, and indicators for inclusion in the provider LDA ≥ 4 output and Figure 8A.

S5. Exploratory Spearman correlations

Spearman correlations were calculated from paired microbiota and phenotype measurements for 18 animals (six per group), pooled across Sham, OVX, and OF44. The experimental team confirmed the animal-level pairing. Table S8b lists anonymized paired animal IDs and the sequencing sample IDs assigned using the author-specified within-group order of Table S3.

Six bacterial families were evaluated against 18 measured phenotypic variables, giving 108 correlations. Spearman ρ was calculated using ranks with average ranks for ties. Two-sided p values were estimated using 99,999 random phenotype-label permutations and the absolute correlation statistic. The plus-one correction was applied as $p = (b + 1)/(99{,}999 + 1)$, where b is the number of permuted absolute correlations at least as large as the observed value. The random seed was 20261020.

BH adjustment was applied across all 108 permutation p values. Table S8a reports ρ, the numerical unadjusted p value, BH-adjusted p, sample size, permutation count, and seed for every pair, including nonsignificant results. Forty-one correlations had BH-adjusted $p < 0.05$. These pooled analyses are exploratory. Associations may reflect treatment-group differences, compositional constraints of relative-abundance data, and unmodeled cage effects. The small sample size and multiple testing limit inference; neither causality, mediation, nor within-group associations are established.

S6. Reporting conventions and software

The term *BH-adjusted p* is used throughout. The adjustment families are: 9 overall alpha tests; 27 Dunn comparisons; 3 pairwise PERMANOVA tests; 3 pairwise PERMDISP tests; 167 hierarchical Kruskal-Wallis tests for the LEfSe input; and 108 Spearman correlations. Statistical significance for these adjusted analyses is defined as BH-adjusted $p < 0.05$. Numerical values are supplied for the complete test families. Cell formatting may round displayed values; workbook numeric values retain their stored precision.

Additional analyses used Python 3.12.14, NumPy 2.3.5, SciPy 1.18.1, scikit-bio 0.7.4, and scikit-posthocs 0.17.1. These versions describe the additional statistical analyses, not the unverified original sequencing-processing environment. Source materials were the OF44 project archive from the provider and the author-supplied individual microbiota and phenotype observations.

Figure S1. Chao1 rarefaction curves

The 18 samples included in the microbiota analyses

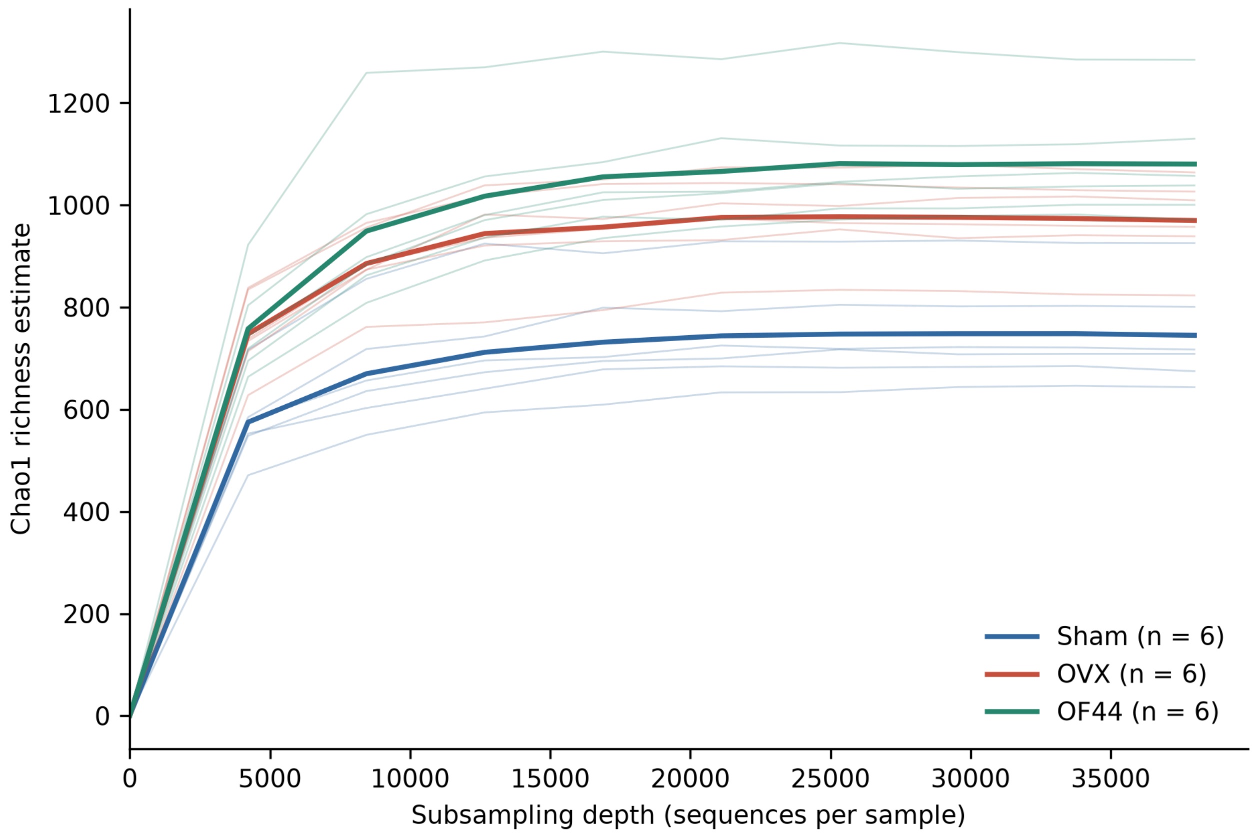

Figure S1. Chao1 rarefaction curves for the 18 analyzed samples from the Sham, OVX, and OF44 groups (6 biological samples per group). Thin lines represent individual samples; thick lines show the group means. Colors identify the three groups. Individual values are the saved provider means across 10 rarefaction iterations at each depth. The available grid extends from 1 to 37,988 sequences; no values beyond this range were extrapolated. The separate analysis rarefaction depth of 39,988 sequences is not a sampled point in these saved curves. Sample-level plotted values are provided in the *Figure S1 curve values* sheet of the supplementary workbook.

Interpretive scope. These curves describe observed Chao1 estimates across the available depth grid. They do not by themselves establish complete sampling of the community or replace the sample-level processing and library-depth information in Tables S3 and S4.
